# Supplementary material for: Locus coeruleus neurons encode the subjective difficulty of triggering and executing actions
Source: PLoS Biol. 2021 Dec 7;19(12):e3001487. doi: 10.1371/journal.pbio.3001487 (PMC8683033; doi:10.1371/journal.pbio.3001487)
Supplement: Attachment — Submitted filename: Bornert et al_response to reviewer.docx [file pbio.3001487.s002.docx]

Dear editors, dear reviewer,

Please find below our response (in bold) inserted in the original review letter.

Best regards,

SB

REVIEWS:

Reviewer #1: The study aims at revealing the relationship between the Locus Coeruleus (LC) activity and effort. The authors recorded single cells activity from the LC in 5 monkeys performing different types of tasks with different levels of reward, in which they either manipulated physical effort (force exerted on a manipulandum - Force-discounting task) or 'cognitive' effort (delay to obtain the reward - delay-discounting task). Finally, in a control task, they used a target detection task to control for sensory-motor constraints (target-detection task). They recorded 75 single units (n=52 in Monkey T and n=23 in Monkey L) in the delay-discounting task, 92 LC units (in 2 monkeys, n=63 in Monkey D and n=29 in monkey A) in the Force-discounting and 26 neurons (in one monkey, Monkey J) in the target-detection task. At the behavioral level, they found that both costs and reward affected the animals' willingness to work and their response times. At the neural level, they foundthat neural activity in LC before and after the go signal carried information about the physical force applied, which exceeded the task parameters. Based on these results, the authors concluded that LC activity reflects the difficulty of initiating an action depending of the context. They also provide an interesting framework in which the LC/NA system might influence effort mobilization.

The question is very interesting and the study is elegantly designed. The results are novel especially the electrophysiological recordings from the LC which are rare. They have the potential to provide key elements to our understanding on how the brain allow us to deal with challenging situations. That said, I have a number of questions and suggestions that I think the authors could address to try and improve the manuscript.

**-> Thank you for this positive evaluation regarding the global interest of our work**

Conceptually and given the question at the heart of the study (relation between LC activity and cognitive and physical effort), it was not entirely clear to me from the introduction why the authors focused their analysis on the action onset and action execution. They should be more clear on which grounds their hypothesis was formulated (page 4, lines 64-66:"We hypothesized that LC neurons would be activated at the time when monkeys did make an effort to face the challenge at hand, both in the cognitive domain (triggering an action that they would spontaneously avoid) and in the physical domain (producing a high level of force)."

The monkeys were familiar with the stimuli and their associated costs and benefits and the cue onset related activity might also carry information about effort, especially given that in the present study, both cognitive and physical effort were manipulated.

**-> This is a very subtle point, but it is indeed crucial, from a conceptual point of view. In short, our hypothesis is related to the relation between LC activity and effort production defined as the *mobilization of ressources* (both in the physical and the cognitive domain). We have modified the introduction (p.3) and discussion (p.37-38, section ‘Behavior’) to clarify this. In particular we now systematically refer to ‘effort production’. In previous studies, we have interpreted the activation of LC at cue onset in terms of mobilization of ressources to process the information provided by the cue (Bouret et al, 2012; Bouret & Richmond, 2015; Jahn et al, 2020), but this remain difficult to prove given the task design (note that we are currently running experiments to address that question…). We modified the discussion (p. 43, second paragraph) to address the issue directly.**

Methods.

- Pages 11 and 12: to identify LC neurons, the authors used different criterion among which "a modulation of firing rate across states of vigilance". Could the authors be more explicit on how this was done? How were the different states of vigilance qualified and/or quantified?
- **-> states of vigilance were observed directly using simple behavioral markers (global level of loco-motor activity, eyes open vs closed and reactivity to sourounding events). This has been added to the Methods section (p.11).**
- Statistical analysis. The authors should provide more details about the statistical analysis carried out in the methods. As is, most of the information are disseminated in the result section and various different statistical analysis were performed such as GLM with model comparison, ANOVAs, t tests first and second order, etc…. For instance, the material in pages 16-18 currently in the result section could introduce the variables of interest and then the authors could outline and justify their statistical pipelines for behavioral and neurophysiological data.
- **We added additional information in the methods (Data Analysis) in order to clarify the variables studied throughout the paper. Additionally, we reorganized this section to highlight the different procedures used. Finally, additional details about the GLMs and model comparisons were added. (p. 15-16)**

Specifically, page 13: "We then fit a generalized linear model (GLM) per monkey on the pooled data for each combination of regressors." can you provide more details about the terms entered in the GLM? How about the random terms (e.g. sessions for behavior and the population analysis, especially when data were not z-scored?) and potential interactions terms?

**-> We added information regarding the models in the methods (see p. 13-14). Practically, we only report results of models including Reward and Delay or Force category since including random terms for the session number did not change the effects of the task parameters, and since the interaction terms were not significant.**

Page 15. They tested different GLM models: "one with only RT as regressor, one with only task parameters as regressors, and one with all task parameters and RT. We used Bayesian Information Criteria (BICs) to compare the fit of the models, but similar results were obtained using Akaike's information criteria (AIC)."

Did the authors statistically compare models with the different terms? Even if BIC and AIC were the lowest, were the model actually statistically compared? were they statistically different? Also, it might be easier for the reader to first have information about the model comparison and then provide the results of the best model.

-> **We are grateful to the reviewer for pointing that out. As he/she probably knows, it is usually admitted that a difference of 3 (or 5) in BIC between 2 models corresponds to a significant difference (since this is a log scale, a difference of 3 units corresponds to a model 1000 times better.). But unfortunately none of this was explicit in the manuscript. We have edited the methods section to explain that principle, along with the description of the procedure for model comparison (p.16). We have also added some explanation in the result section (p.29, last paragraph; p.31, last paragraph).**

Results and discussion.

- In the Force-discounting task, there were 3 different levels of force and it is stated that "the force had been maintained above threshold". Shouldn't this also have an upper limit threshold (looks like it from fig S1)? This could be added in the methods.
- **-> we did not include an upper limit threshold because this introduces another difficulty (precision) which dramatically changes the relation between the required force level and the difficulty (and therefore the effort required to perform correctly). We added a sentence to specify that there was no upper threshold in the methods (p. 10, 1st paragraph).**
- In this same task (but not in the others), the monkeys had to maintain fixation, which adds 'effort mobilization' to the task? How do they authors think this contributed to the neural activity in the LC? It might be worth discussing this issue.

**> This is an interesting issue, but unfortunately, we cannot address it here. Fixation presumably induced a global increase in task demand and therefore a global level of effort, but it is hard to verify since we do not have a proper reference. Since evaluating the absolute level of effort is actually challenging, virtually all of our conclusions here are based on parametric modulations, i.e. on the difference in effort level across conditions. Typically, we do not believe that the control Target Detection task involves no effort production at all, but rather that the contrast in the amount of effort produced across conditions is negligible. By analogy, we would need to compare behavior and LC activity in a version of this task that does not require fixation, and unfortunately we do not have the data. We have edited the discussion to address this point (e.g. p. 39, end of the 1st paragraph).**

* The results at the population level nicely support a relation between LC activity and subjective effort in both cognitive and physical domains. Yet, when considered individually, few neurons were significantly modulated regardless of the task. Do the authors think this tells us something about the coding within LC neurons? How so? I think this disserves some discussion.

**> indeed, this is an important point, and we have added a short paragraph in the discussion to address it. In short, this implies that the information is shared by a large number of LC neurons, even if its influence on firing remains small for many of them. We have proposed that this information is provided by global inputs from the PGi, which innervates most LC neurons (see bottom of p. 42, top of p. 43.**

- I am also wondering whether the direction of the effects (increase or decrease of activity) were informative more so at the action onset since this seems to be more related to the type of action during action execution?

**-> We are not sure to understand that statement. The direction of effect is very informative in any case, and there is only one type of action in each of the task. What we show here is that right before action onset, the activity of LC neurons is modulated by RT (over and above task parameters), but not by exerted force. By contrast, during force production, LC activity is modulated by force production but not by RT (again, after removing the influence of task parameters). But this is indendent upon the direction of effects.**

* I suspect that the subjective effort varied across the sessions and I understand that this might not be evident to assess these subtle changes on top of the other effects but could the authors detect such evidence looking at the effect across time and actually in any time window?

**-> This is an interesting point: we examined this in previous studies, and the effect was variable. Fore example, in another task, the negative influence of reward on LC activity at action initiation increased (i.e. firing decreased more with increasing rewards) as monkeys advanced in the session and their motivation decreased, in line with an interpretation in terms of cognitive effort to trigger the action (Bouret & Richmond, 2015, fig 8E&F). Here, we could not find any effect of trial number on spike count, nor did it have any significant interaction effect with the other parameters. Thus, trial number was not included in our models.**

- The authors found that "The activation before action onset was especially pronounced in discounting tasks, where triggering the action could involve some form of cognitive effort. (Page 25)." Is that possible that the lack of effect in the target detection task at the population level might be related to lack of statistical power (fewer neurons sampled from only one animal)?

**-> this should clearly be taken as a possibility, and we added this in the discussion (p. 41, last paragraph). But it is unlikely, because the effect size if smaller in the target detection task, such that for a population of that size in the discounting task, the effects would appear significant. Although we did not test for that systematically, we replicated the analysis with a random sample of 26 neurons from the Delay-discounting task and from the Force-discounting task and the significance of the coefficients for task parameters and RTs remained, suggesting that in these samples 26 neurons sufficed to detect effects (see result section, last paragraph on p. 32).**

- Page 20; line 383, please report beta coefficients.

**-> yes, thank you**

* Page 26, please indicate time window

**-> yes, thank you**

Reviewer #2: This is a fascinating paper by an established team of primate electrophysiology researchers. They have recorded in the locus coeruleus, and link the responses of neurons there to production of effort. Their analyses are focused on the question of whether firing rates in these neurons covaries with effort. They make use of a well designed set of three tasks, and on careful analysis of behavior, to establish the variables they are interested in.

The chief appeal of this paper is its focus on the LC, which is difficult to record from. As a result data from this region are lacking in the literature. As such, this report is likely to have a good deal of influence. And LC is not simply an obscure region - it has a great deal of importance for a great deal.

One of the strengths of this paper is the care that the authors put in to thinking about effort - they use a WTW measure, a reaction time measure, and a behavior measure. This, combined with careful line of thinking about how each of these variables interacts, combine to give them a good deal of insight into the deployment of effort.

**-> Thank you for this positive evaluation regarding the global interest of our work**

I would consider the following comments to be minor ones:

"But still, monkeys failed to initiate the action more often in trials associated with higher cost and/or lower rewards, which indicates that they could not repress a natural tendency to disengage in such conditions, even if in that situation if was counterproductive." This seems like suspect logic to me. I agree this is suggestive, but there would seem to be other explanations for these effects. It could also be there is no natural tendency, but instead monkeys engage in a natural

**-> Indeed, there could be other interpretations, and we certainly acknowledge that in the manuscript. The end of the sentence is missing, so we are not sure of what the reviewer means, but another account of this behavior could be that monkey simply engage in the task as a function of the expected outcome value. But still, even in such a scenario, since this is suboptimal in terms of reward rate, it remains compatible with the existence of a complementary effect to counter this tendency to not engage in trials with relatively low value, to try to get closer to optimality (and increase the reward rate). We address this issue in the discussion (p. 38, second paragraph).**

The authors seem to be supporting a model in which the cost of various cognitive / physical behaviors, in terms of effort, are constant, and willingness to pay is what varies. It seems to me that the opposite is also possible - the subjective cost may vary and the willingness to expend effort may stay the same. Or alternatively, both may vary and we dont known which is which.

**-> We assume that the reviewer refers to willingness to work, rather than willingness to pay and/or willingness to expand effort. Here wilingness to work (wether monkeys are willing to perform a trial given the task conditions) is evaluated using engagement rate, which varies as a function of expected costs and benefits associated with distinct task conditions. And we assume that the costs and benefits associated with task conditions are relatively stable, because they correspond to objective variables (delay, reward size, physical force to be exerted). We tried to address this issue both in the methods (p.12) and in the result section (p. 17, 2nd paragraph).**

"Thus, we assumed that in task conditions associated with lower willingness to work, engaging in the task and performing the action required a higher level of cognitive control to overcome the stronger tendency to disengage, compared to conditions in which average willingness to work was higher." What if the reverse is true - subjects wait until the cost is lower to do it? Then LC would mean something else.

**-> We do not understand what the reviewer means here, because if monkeys wait until the cost is lower, i.e. refuse to perform the offered option, then there is no behavioral response and no LC activation to interpret. And this is perfectly in line with our interpretation, in a way, even it is quite difficult to interpret an absence of response. In any case, the LC is more active in conditions where, on average, monkeys tend to engage less, because the cost (delay or force) is higher and/or because the benefit (reward size) is lower. So wether it is described as a stronger tendency to disengage or as a weaker tendency to engage, it remains that LC neurons are more active in these conditions when monkeys need to bridge a larger gap between not-engaging and engaging. And our claim is only about the amount of cognitive effort needed to bridge that gap. We assume that this is related to the first remark of the reviewer, which we addressed in the discussion (p.38).**
